# Invasive fungal disease in PICU: epidemiology and risk factors

**DOI:** 10.1186/2110-5820-2-6

**Published:** 2012-02-22

**Authors:** Olivier Brissaud, Julie Guichoux, Jerome Harambat, Olivier Tandonnet, Theoklis Zaoutis

**Affiliations:** 1 Pediatric and Neonatal Intensive Care Unit, The Children's' Hospital of Bordeaux, Place Amélie Raba Léon, 33076 Bordeaux Cedex, France; 2 Division of Infectious Diseases, The Children's Hospital of Philadelphia, CHOP North Room 1527, Philadelphia, PA 19104, USA

## Abstract

*Candida *and *Aspergillus spp*. are the most common agents responsible for invasive fungal infections in children. They are associated with a high mortality and morbidity rate as well as high health care costs. An important increase in their incidence has been observed during the past two decades. In infants and children, invasive candidiasis is five times more frequent than invasive aspergillosis. *Candida sp*. represents the third most common agent found in healthcare-associated bloodstream infections in children. Invasive aspergillosis is more often associated with hematological malignancies and solid tumors. Recommendations concerning prophylactic treatment for invasive aspergillosis have been recently published by the Infectious Diseases Society of America. *Candida albicans *is the main *Candida sp*. associated with invasive candidiasis in children, even if a strong trend toward the emergence of *Candida *non-*albicans *has been observed. The epidemiology and the risk factors for invasive fungal infections are quite different if considering previously healthy children hospitalized in the pediatric intensive care unit, or children with a malignancy or a severe hematological disease (leukemia). In children, the mortality rate for invasive aspergillosis is 2.5 to 3.5 higher than for invasive candidiasis (respectively 70% vs. 20% and 30%).

## Introduction

*Candida *and *Aspergillus spp*. are the most common agents responsible for invasive fungal infections (IFI) in children. They are associated with a high mortality and morbidity rate as well as high health care costs. Their incidence has dramatically increased within the past two decades [1-3]. In children, invasive *Candida *infection (ICI) is five times more frequent than invasive *Aspergillus *infection (IAI). *Candida spp*. is the third most common agent implicated in healthcare-associated bloodstream infections in children [4-8]. IAI is more often associated with hematological malignancies and solid tumors. Strong recommendations concerning prophylactic treatment for IAI have been published [9]. Although *Candida albicans *is still the main *Candida sp*. associated with ICI in children, a strong trend toward the emergence of *Candida *non-*albicans *has been observed. This could be linked to the use of fluconazole prophylaxis in some patients [2,4]. The epidemiology/risk factors for IFI are quite different between previously healthy children hospitalized in the pediatric intensive care unit (PICU) and children whose hospitalization is related to malignancy or a severe hematological disease (leukemia). Indeed, in the second group, the reported incidence is approximately 5% with a mortality rate of approximately 60% [10]. The crude mortality of patients with IFI is 32% [11]. The mortality rate due to IAI in children is approximately 70% despite appropriate treatment, whereas it is between 20% and 30% for ICI [5,12].

### Fungal infections and nosocomial infections

In France, a 1-day national survey of nosocomial infections performed in 2001 included 21,596 children younger than aged 18 years (7.1% of all hospitalized children) [13]. Overall, 2.4% presented with a nosocomial infection (1.2% for newborns and 3.3% for children). ICI, in this study, accounted for 4.4% of all infections, regardless of which unit the children were hospitalized in. The rate of nosocomial infections in children hospitalized in PICUs was approximately 15% [13]. Posfay-Barbe et al. reported a *Candida *infection rate of 10% in children younger than aged 18 years in the United States, regardless of age and hospital unit [11]. In 1999, Richards et al. [8] showed that *Candida spp*. was implicated in 9.4% of bloodstream infections in PICUs in the United States. An Israeli study [14] found *Candida spp *in 14.4% of bloodstream infections in PICUs. *Aspergillus *was found in 0.5% of lung infections and 0.1% of sepsis [8].

### Invasive candidiasis in the PICU

#### Epidemiology

ICI in the PICU presents as candidemia or disseminated candidiasis (kidney, liver, eye, etc.). In 2000, a large U.S. study on the epidemiology and outcome of hospitalized adults and children with candidemia showed a higher candidemia rate among hospitalized children than adults (43 vs. 30 cases per 100,000 admissions, respectively) [7]. Dutta et al. reported an increase in the incidence of candidemia in their institution (Houston, Texas), rising from 0.06 to 0.3 per 1,000 inpatients from 2000 to 2009 (with an increase from to 2000 to 2004 followed by a stabilization after 2005) [15].

ICI is a very severe disease with an attributable mortality in children between 20% to 30% [5,12], and a mortality rate among children with ICI between 16% to 31% [5]. Depending on the study, *Candida *is either the second, third, or fourth causative agent for sepsis in hospitalized children, after coagulase negative *Staphylococci*, enterococci, and *Staphylococcus aureus *[4-8]. Singhi et al. reported recently that *Candida spp *colonization occurs in approximately 70% of pediatric patients in the PICU [16]. The risk of colonization is particularly important in small children [17]. In the report by Zaoutis et al., the incidence of candidemia in the PICU was 3.5 per 1,000 admissions [3].

*Candida albicans *is more predominant in children than in adults. Moreover, *Candida albicans *remains, in various studies, the most common fungal agent associated with ICI regardless of age (55%), followed by *C. parapsilosis *(17.5%), *C. tropicalis *(10%), *C glabrata*, and *krusei *(2-3% each) [4]. Although *Candida *albicans is the main *Candida *species in children, some strains are associated with specific conditions: *C glabrata *among surgical patients or those with a central venous line; *C. tropicalis *among patients with malignant disease or neutropenia; and *C. parapsilosis *among infants or those on parenteral nutrition.

Pfaller [18] reported data on blood samples from 79 medical centers throughout the world and confirmed these results in 256 children aged 0 to 19 years: 50% of patients experienced infection with *C. albicans*, 28.5% with *C. parapsilosis*, 12.9% with *C. tropicalis*, 2% with *C. glabrata*, and 0.8% with *C. krusei*.

#### Risk factors

Hospitalization in the PICU represents a risk factor for ICI. Other risk factors include presence of a central venous line, parenteral nutrition, preexisting bacterial infection, immunocompromised status, recent surgery, dialysis, prolonged use of vancomycin, and mechanical ventilation [6]. Table 1 summarizes the risk factors for developing ICI in children hospitalized in the PICU. These risk factors can coexist. The role of the central venous line in the occurrence of disseminated candidiasis is well known; it increases threefold the risk for developing a disseminated infection (odds ratio [OR] = 3.0; 95% confidence interval [CI], 1.2-7.8; *p *= 0.02) for patients who have a central venous line in place for 3 days [19]. The recommendations given by the Infectious Diseases Society of America are very clear regarding central line replacement in case of ICI: "Central venous catheters should be removed when candidemia is documented, if at all possible"[9]. Predicting the risk for children to develop candidemia in the PICU poses an important challenge. By combining various risk factors in 101 children hospitalized in the PICU, it has been shown recently [3] that the risk of ICI in this population increased from 10% (malignancy + central venous line + more than 3 days of vancomycine) to 46% (antibiotics with anti-anaerobic activity + malignancy + central venous line + total parenteral nutrition + more than 3 days of vancomycine). Based on data from studies in adult patients, some authors have suggested using antifungal prophylaxis in patients for whom the risk of candidemia is higher than 10% [20].

**Table 1:** Risk factors for Invasive *Candida *Infection in children (from [3])

Risk factors for invasive candidiasis	Unadjusted odds ratio	95% Confidence interval
Malignancy	3.22	1.36-7.6
Presence of a central venous catheter	13.4	4.8-37.42
Presence of a arterial catheter	1.77	1.02-3.06
Receipt of total parenteral nutrition	5.3	2.8-10.05
Neutropenia within 15 days	5.58	1.12-27.79
Non-candidal blood stream infection within 15 days	2.47	1.35-4.52
Receipt of antifungal agents within 15 days	2.86	1.44-5.66
Receipt of antibiotics within 15 days	5.44	1.87-15.77
Parenteral or oral vancomycin during 1-3 days	2.56	1.27-5.16
Parenteral or oral vancomycin ≥4 days	3.17	1.73-5.82
Extended-spectrum cephalosporins ≥4 days	2.31	1.26-4.22
Carbapenems ≥4 days	3.29	1.1-9.89
Aminoglycosides	2.09	1.17-3.74
Agents covering anaerobic organisms ≥4 days^a^	2.3	1.29-4.11

^a^Including ampicillin sulbactam, clindamycin, imipenem, meropenem, metronidazole, and ticarcillin-clavulanate

There is an association between ICI and *Candida *colonization. Singhi et al. [16] noted a positive correlation (*p *= 0.033) between colonization and candidemia among PICU patients, regardless of site of colonization. Colonization was four times more likely in patients with a central venous line. Authors compared 20 patients with candidemia and 45 patients without candidemia. Eighteen of the infected patients were colonized with *Candida *(90%), whereas only 27 of the noninfected patients were colonized (OR = 6; 95% CI, 1.2-29.1; *p *= 0.03). In the literature regarding adults, many publications have reported scores or indexes based on localization of *Candida *colonization, and the presence or not of clinical signs, to identify people at risk of developing ICI [21-23]. Pittet et al. showed the value of using a *Candida *colonization index (ratio of the number of distinct, non-blood, body sites, screening positive for *Candida spp *to the total number of distinct body sites tested) to identify colonized patients who should be treated preemptively with antifungal therapy [23]. The authors chose a cutoff value ≥0.5 for their index. Although the sensitivity of the proposed index was high (100%), its positive predictive value was poor (66%). Patients enrolled in the study by Pittet et al. were all from surgical units. It is possible that this colonization index is more useful in a unit with a known high incidence of candidemia. In units with a low incidence rate (medical ICU for example), associating clinical or/and biological signs with this index could enhance its accuracy. It would be legitimate to take a similar approach in children to offer more reliable, early preemptive or probabilistic treatments. Such studies in children are not currently available and should be done. Lortholary et al. reported a risk of being infected with a strain presenting reduced susceptibility to an antifungal therapy (Caspofungin or Fluconazole), in cases of recent exposure to one of these two drugs [24].

#### Mortality

**Figure 1:**
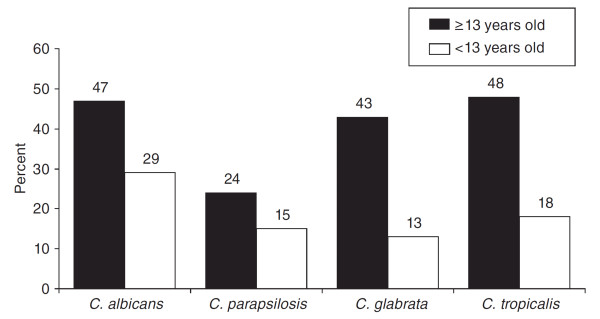
**Mortality associated with type of *Candida spp*. among patients with candidemia younger (n = 144) and older (n = 1,447) than 13 years **[6].

In children, candidemia was associated with a 10% increase in mortality (95% CI, 6.2-13.8%), a 21.1-day increase in the average length of stay (95% CI, 14.4-27.8 days), and an average increase in total hospital costs of $92,266 per patient (95% CI, $65,058-119,474) [7]. The mortality rate for candidemia was 30% for children and reached 43-54% in infants [7]. As shown in Figure 1, the mortality rate due to *Candida *infection is highly correlated to the species of *Candida *and the child's age [6]. Overall, it should be noted that mortality rates appear higher in older patients regardless of the strain involved. Some authors reported that ICI linked to *C. parapsilosis *appeared to be less aggressive than cases linked to *C. albicans *(27% vs. 47% mortality rate in children younger than 13 years; n = 144) [6]. However, Dutta et al. [15] showed, in a series of 108 children, no difference in terms of 30-day mortality between strains of *Candida*. Hospitalization in the PICU at the time of diagnosis of invasive candidiasis and the presence of an arterial catheter were independent risk factors for increased mortality [25]. High mortality rates have been observed in specific populations, and some authors have identified ethnic origin as a possible risk factor for developing ICI, especially in Filipino patients [26]. No other studies have confirmed these data.

#### Candidiasis and children in pediatric oncology

In pediatric oncology units, there often are more yeast infections than mold infections. In the study by Mor et al. [10], concerning 75 children with IFI and malignancy pathologies, positive blood cultures for yeast were obtained in 100% of infected cases. The main underlying diagnoses associated with yeast infections were acute myeloid leukemia (AML) and neuroblastoma (26.7% each); five patients (older than aged 15 years, with invasive yeast infections) underwent hematologic stem cell transplantation (HSCT). Sung et al. [27] performed a survey of the available data from the pediatric cancer population (more specifically patients with AML) and IFI. They reported that during the induction and consolidation phases of treatment, respectively, 10% and 6% of children developed *Candida *infection. In this study, *Candida spp*. represented 25.9% of infection-related mortality (overall 11% ± -2%). The authors observed that in the case of intensive timing induction phase, compared with standard timing induction, the rate of yeast infections can increase 2.5-fold (8.4% vs. 19%; *p *< 0.01). So, the timing of the chemotherapy, regardless of dose, has an impact on the patient's risk for developing IFI. In children hospitalized in the PICU, the existence of an underlying malignancy is very important and increases from 17.5% to 46% the predicted probability of ICI [3].

### Invasive *Aspergillosis *infections in PICU

There is little specific data available in the literature regarding children with IAI [5]. Furthermore, the available data are not homogeneous (definition, incidence, concerned population) making analysis difficult. Clear incidence of IAI in children with or without a hematologic underlying disease, or in the PICU, is not easy to find. Some authors report the overall incidence, others the incidence in a specific subpopulation, whereas other papers give the proportion of children with IAI in specific conditions, i.e., presence of an underlying disease (such as AML or acute lymphoid lymphoblastic, for example). Another limitation is that definitions and care have changed over the decades. The largest study concerns 139 cases of IAI in children and only reports data until July 1, 2005 [1].

#### Epidemiology

Studies report a three- to fourfold increase in the incidence of IAI during the past decade [5,28], mainly attributable to the improvement of the quality of treatment and the survival rate of immunocompromised patients. In 2006, the annual incidence of IAI in children was 0.4% in the United States. Three quarters of these patients were immunosuppressed or suffered from oncological diseases [5]. In 2008, Crassard et al. [29] published a series of 24 cases of proven or probable IAI in children hospitalized in a pediatric hematologic department during a period of 15 years (1986-2000). Interestingly, the median interval between the onset of the malignancy and the diagnosis of IAI was 8.5 months. Fifty percent of infected patients had ALL or AML. Ten children underwent HSCT before the occurrence of IAI. Overall, 15 children (62.5%) died. The mortality attributable to IAI was estimated to be 37.5% (9 children), 60% of overall deaths. The incidence of IA varied according to the underlying disease: 5.35% in AML, 1.5% in ALL. Other studies reported the same variation in incidence of IAI according to the underlying disease: 4% in AML and 1% in ALL for Abbasi et al. [30], and 3.7% in AML and 0.6% in ALL for Zaoutis et al. [31].

**Figure 2:**
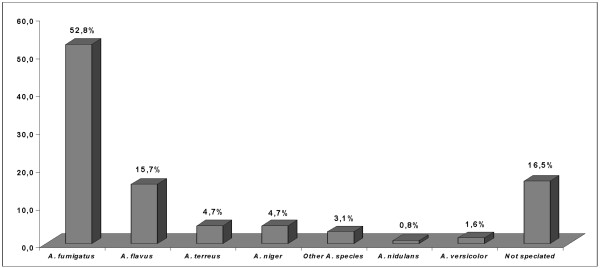
***Aspergillus *species encountered in invasive aspergillosis in 139 children **[1].

There are conflicting results in the literature regarding the most commonly identified species of *Aspergillus *in pediatric IAI. The most common strains observed in the pediatric population are *A. fumigatus, A. nidulans, A. flavus, A. terreus*, and *A. niger*. The largest contemporary study reporting IAI in children showed, as reported in adults series, *A. fumigatus *as the first causative agent in this disease (Figure 2) [1]. *A. flavus *was considered as the first causative organism in two previous studies [32,33]. In children, *A. fumigatus *seems to be the most common species encountered in the pulmonary form of the disease. *A. flavus *is predominantly found in skin infections [1]. Specific consideration must be given to *A. niger *whose incidence can reach 6.5% in septic granulomatous disease [31].

The most frequent localization was the lung, ranging from 59% to 91.6% of infections [1,30,33,34]. Walmsey reported predominance of up to 41% for IAI in skin lesions [33]. However, this was not confirmed by others publications, which found respectively 10% [1] and 20% [30] of cutaneous aspergillosis.

**Figure 3:**
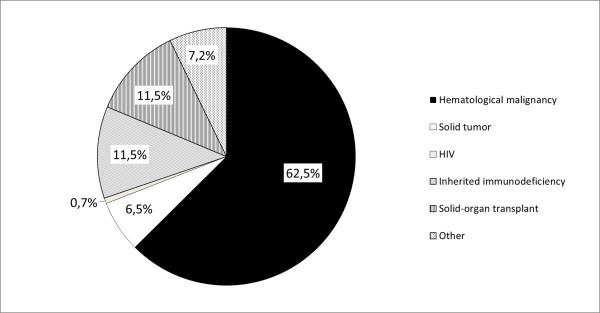
**Underlying condition of the 139 cases of invasive aspergillosis in the study by Burgos et al. **[1].

**Figure 4:**
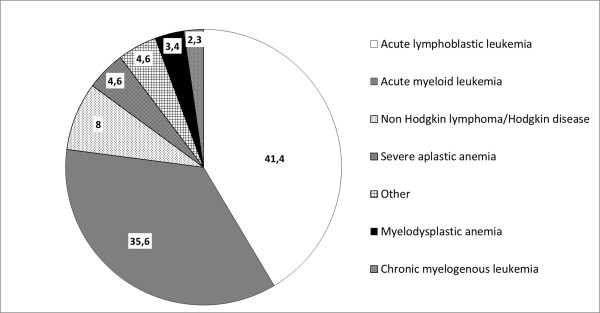
**Details of the 87 underlying haematological disease in children with invasive aspergillosis in the study by Burgos et al. **[1].

More anecdotic localizations (but not necessarily less dangerous or difficult to treat) have been described: cerebral, tracheobroncheal, renal, bone, endocardial, blood, and eye. Dotis et al. performed a systematic review of the literature for children with central nervous system *Aspergillosis *[35], identifying 90 cases since 1950. When considering children of 1 year or more, leukaemia (ALL statistically more frequently than AML) was the predominant underlying condition, followed by solid tumors, liver transplantation, chronic granulomatous disease, other hematological disorders, and various other conditions. Figures 3 and 4 summarize the underlying conditions encountered in the 139 cases of IAI reported by Burgos et al. [1]

#### Risk factors for IA

Risk factors for IAI are well known in both the adult and pediatric population. It is fundamental to understand that immunocompromised children can contract community- or hospital-acquired infections (source: construction work, renovation, air conditioning system contaminated). Risks factor include presence of several underlying diseases and their treatments,: hematologic malignancies, either primary or relapse, allogeneic bone marrow transplantation [34]; granulocytopenia, corticosteroids for malignancy but also autoimmune disease, immunosuppressive therapies, immunodeficiencies, such as chronic granulomatous disease, severe combined immunodeficiency, and organ transplantation, such as heart-lung transplantation [36]. There is evidence in the literature that the risk of IAI increases with higher doses of steroids (most often used in bone marrow transplantation) notably beyond a dose of prednisone > 2 mg/kg/j [34]. Cushing syndrome because of its endogenous high secretion of cortisol can favor development of IA [36]. Some risk factors, such as persistent neutropenia, CMV infection, prolonged antibiotic administration, viral respiratory infection, HIV infection, and prior colonization with *Aspergillus*, are confounding factors with hospitalization in PICU or with the underlying malignancy itself [28]. Patients with AML and relapsed ALL are particularly at risk for IAI [31].

#### Mortality

Within the past decade in the United States, there has been a 357% increase in deaths related to IAI, regardless of the patient's age [34]. Risk factors for overall mortality in pediatric patients with IAI are summarized in Table 2. Patients with IAI have a 20% increase in mortality rate, a 13.5-fold increase in relative risk for death. Paediatric patients with IAI had a longer median length of hospital stay than immunocompromised children without IA (respectively 16 days and 3 days) [31]. Even with appropriate treatment, therapeutic response to IAI rarely exceeds 50%. In the study by Burgos, the mortality rate for treated patients reached 52.5% [1]. Lin et al. [37] reported an overall IAI mortality rate of 58% when including patients of all ages and 68% in patients younger than aged 20 years. The mortality rate varied according to the underlying disease: 88.1% in cases of disseminated aspergillosis or CNS involvement, 86.7% in cases of bone marrow transplantation, 85.7% in HIV infection or AIDS. Highly active antiretroviral therapy (HAART) has profoundly transformed the incidence and prognosis of IAI in patients with HIV and AIDS.

**Table 2:** Mortality risk factors in invasive aspergillosis (from [1])

Conditions	Alive (n = 66)	Dead (n = 73)	*p*
Bone marrow transplantation			0.001
Autologous	1 (2)	1 (1)	
Allogeneic	11 (17)	40 (55)	
Graft-versus-host disease	3 (5)	20 (27)	0.01
Corticosteroid treatment	42 (64)	62 (85)	0.033
Immunodeficiency	22 (33)	47 (64)	0.001
Surgery after diagnosis	38 (58)	23 (32)	0.045

Data are numbers with percentages in parentheses unless otherwise indicated

The overall mortality rate for children with AML and ALL is quite low (respectively 3% and 1%). However, if children with AML or ALL develop IAI, the mortality rate increases 5-fold for AML and 14-fold for ALL (respectively to 20% and 21%) [5,31].

CNS aspergillosis-related mortality is usually reported as exceeding 80% [37]. However, in the study by Dotis et al. [35], the overall mortality of cases published in the literature since the 1950s was 65.4% with a strong difference between the periods before and after 1990 (respectively 82.8% vs. 39.5%). This may be due to improved patient care. Table 2 summarizes the risk factors for mortality in IAI identified by Burgos et al. [1].

## Conclusions

The frequency and severity of IFI in children has increased steadily during the past 20 years. This is due to the higher prevalence of susceptible hosts who are kept alive with more aggressive therapy (cytotoxic, immunosuppressive), the specific treatment of hematopoietic stem cell transplantation, and the use of broad-spectrum antibiotics. *Candida spp*. and *Aspergillus spp*. are the most frequently identified fungi in children. The attributable mortality of each of these two invasive infections remains different mainly because the affected patients are different (more hematologic malignancies in patients with *Aspergillus *infection). Preemptive therapy has been well defined in cases of IAI, and recommendations have been provided by the IDSA [9]. The real challenge remains to identify patients with a high risk of invasive candidiasis in the PICU to propose targeted prophylaxis.

## Competing interests

The authors declare that they have no competing interests.

## Authors' contributions

TEZ has participated in the design, the editing, the drafting of the manuscript and language corrections; JG participated in the drafting, the proofreading and corrections of the manuscript, JH participated in the drafting, the proofreading and corrections of the manuscript, OT participated in the drafting, proofreading and corrections of the manuscript, OB participated in the design, the editing, the drafting, proofreading and corrections of the manuscript. All authors participated for the literature review, read and approved the final manuscript.
